# Abstracts and Selections

**Published:** 1912-12

**Authors:** 


### Abstracts and Selections.

Young and Sherwood, from the University of Kansas, in the *Journal of Industrial Engineering Chemistry*, have shown that the bacteria artificially introduced into carbonated waters and in plain waters containing the various flavoring syrups are not completely and entirely killed even after long periods of exposure. They introduced various micro-organisms such as B. coli, B. typhosus et al. into the various summer drinks as soda water, pop, etc., subsequently testing these drinks to determine if there had been any bactericidal action. The findings of these investigators are of interest in view of the fact that the public and some physicians generally believe that the summer beverages are absolutely healthy and even the manufacturers hold the view that during the process of making, all bacteria are destroyed. It is evident that water of uncertain bacterial content should not be used in preparation of the various carbonated drinks without proper and efficient sterilization or filtration.—*Ohio Bulletin.*

---

The Texas Sanitarium, at Llano, Texas, an institution devoted to the treatment of tuberculosis, has been under process of reorganization, and it now has more than three-fourths of this organization completed. More prominent physicians are associated with this organization than any institution ever started in the Southwest. The organization will be completed by the end of the month. This means the success of the institution and its enlargement along many lines. It is the purpose of those now interested to influence the building of memorial cottages upon very liberal conditions. Details of the plan will be furnished at a later date.

---

A history of discomfort and oppression in the chest and throat after eating, relieved by induced vomiting, suggests cardiospasm.—*American Journal of Surgery.*

---

Lady Frances Cook, pioneer in the woman suffrage movement, now nearing the seventieth milestone, is among the most active of those who are promoting eugenic principles in England. In expressing her views on the subject, Lady Cook said: "I do not agree with those eugenists who would sterilize the unfit. We must make sure that only the fit are allowed to be born, and that can only come by inculcating a reverence for fatherhood and motherhood; not by foolishly allowing children to grow up, acquiring their knowledge of 'the temple that was not built with hands'—the body—in a promiscuous manner. Then you would have your streets filled with degenerates and the unemployed. I believe in early marriages. Let the men sow domestic oats instead of wild oats. There are thousands of men that know that their mothers are their best friends, and those are the sort of men we want. When we can get enough of them, the woman question, as it is called, will be solved; for no true man believes for a moment that his mother, his wife or his sweetheart is one whit less capable of playing her part in the world than he is."—Exchange.

Note by editor of "Red Back."—Lady Cook, of London, was "Tennie" Claflin, of America, sister of Victoria Woodhull, once a candidate for President of the United States. She is now Lady Martin, of London.

